# Physiology

**Published:** 1825-07-01

**Authors:** 


### Quarterly Periscope

OF

### PRACTICAL MEDICINE;

BEING

### The Spirit of the Medical Journals,

*Foreign and Domestic;*

WITH COMMENTARIES.

### I.

### Physiology.

——————— latas à numine leges

Religiosa doce, mentesque cupidine veri

Allice.

1. *Vivisections.* We need hardly remind our readers that we have uniformly reprobated the extravagant rage for vivisections now pervading many parts of the continent, more especially France. But in so doing, we as uniformly maintained that experiments on animals, when carried on for the purpose of elucidating problems in Physiology, Pathology, or Therapeutics, and conducted with all possible care to avoid unnecessary cruelty, were justifiable. In this country vivisections are so exceedingly rare, that we could not but consider the "*no cruelty*" cry (a worthy successor to "*no Popery*") which has been lately raised by a party in the profession, as neither more nor less than a hypocritical cant to curry favour with the extra-professional saints, and thus promote their own private interests. The violent rage engendered by this expression of ours, among these conscientious *Medico-Martinets*, may be estimated by the following epistle from a town that has long flourished by the blood which followed the stripes of the Negro-driver!

"*Liverpool*, *29th April*, 1825.

"Sir,—I cannot resist addressing you as the avowed advocate of *cruelty* and *inhumanity*, and were I personally acquainted with you I should 'cut you'—*direct.* In your quarterly Journal, I read it to be *hypocrisy* in the medical men to abstain from cruelly torturing animals, for (as you would term it) useful purposes. Let me ask what has the injection of urine as performed by Blundell, done for the good of society. We all know how you would squeak if one drop should get between the coats of your intestines, but we should not regret the consequence, to such a monster. I say it is laudable, that men professing humanity, should shew the world, that unnecessary experiments are abhorred by them (you must know we have bad name enough for that.) When it is lawful, pray let it be sanctioned by others, and not that every whipster should plunge a blunt knife into the chest of an animal, to see which way the blood flows. Lastly, you will find a deficit somewhere, scratch my name (the name of an anonymous dastard!) out as a subscriber to your Journal, in many respects a valuable one, and if you continue to hold up such doctrine, I shall continue to believe you some *dog-butcher*, and unworthy the name of a physician."

This is the language of men affecting such delicacy of feeling, and scruples of conscience, that they shudder at the inhumanity of pushing a needle through the web of a frog's foot—while they can lick their lips and stroke their paunches at the sight of eels skinned *alive*, and afterwards fried *alive*—fish carped *alive*—lobsters boiled *alive*—oysters stewed *alive*, or eaten *alive*, &c. &c. &c.! "Oh," they will say, "this is *necessary* cruelty." Do we not, before sitting down to a table covered with these victims, turn up our eyes and cry—"Lord bless *these creatures to our use*, and us to thy service, &c." We do like consistency—and therefore maintain that the advocates for sparing the lives of animals from the surgeon's knife, should also restrain the hand of the butcher. The life of the animal is sacrificed, in the one case, for the sake of science, and for the ultimate good of the human race—in the other, for the sake of pampering the appetite, and laying the foundation of innumerable diseases.*

This "no cruelty" cry is levelled at the memory of the illustrious dead, and the moral character of the meritorious living. Among the experimenters on animals, with scientific views, are almost all the names that grace and honour our profession. A Galen, a Harvey, a Haller, a Spalanzani, a Hunter, a Parry, among the dead—A Cooper, a Brodie, a Bell, a Philip, and many other distinguished names among the living, of our own country. These are the men whose memories are to be aspersed, and characters assailed by a sect of canting hypocrites who, having neither zeal nor talents themselves, endeavour to gain popularity by vituperating their superiors.

We say again, we like consistency. We could not, therefore, but be amused while reading a long medical sermon, in the newspapers, from the NIMROD of Dublin, on *cruelty to animals!*—The man who had no sooner finished his homily on the inhumanity of pithing a *rabbit*, than he mounted his courser and hunted to death an innocent *hare!* The argument cannot be upheld that we are compelled to eat animal food.

* We venture to affirm, without fear of contradiction, that there is more physical torture inflicted, and mental agony occasioned, among animals, in one winter, in England, by hounds and guns, than during two thousand years by physiological experiments. Yet the former are looked upon without any other emotion than that of pleasure, while the latter are held up as instances of unparalleled inhumanity! Such is the *consistency* of the Pseudo-philanthropists.

Whole nations, as the Hindoos, live on vegetable substances, and enjoy good health. The Irish peasant seldom tastes animal food, and is remarkably robust. If therefore, we wish to respect the lives and feelings of the animal kingdom, let us turn to vegetable diet. But what excuse can be offered for the conduct of the sportsman, who wantonly destroys myriads of the most innocent animals—while, like the tiger, he can only make use of a few ounces himself. Yet these are the men who raise the "no cruelty" cry against the physiologist for making a few solitary experiments on the lower animals, with the express design of saving human sufferings.

We repeat what we have often urged in this Journal, that we reprobate the indiscriminate and senseless experiments of some of the continental physicians; but we do maintain that the interested cry against physiological experiments in this country (where they are proverbially rare) is a disgrace to those who have raised it—and we shall never cease to tell them so, be they high or low in the profession.

---

2. *Pneumo-gastric Nerves—Digestion.* M. M. Breschet and Edwards have published in the ARCHIVES for February last, a memoir on the mode of action of the abovementioned nerves in the production of the phenomena of digestion. The experiments are too long and too numerous for analysis here. We can only give the conclusions to which the experimenters have come. They are, substantially, as follows:—

1mo. The division of the par vagum considerably retards digestion, but does not arrest that process.

2ndo. This retardation of the digestive process depends upon defective action of the muscular coat of the stomach.

3tio. The vomiting which often follows the section of these nerves, is owing to paralysis of the muscular fibres of the œsophagus.

4to. The re-establishment of the digestive process, after the division of the nerves in question, by means of electricity or galvanism, depends —not upon the chemical action of this agent, but on its exciting the movements of the muscular coats of the stomach necessary to the renewal of the surface of the alimentary mass, and to the placing successively all the parts composing it in contact with the parietes of the stomach.

5to. By means of a mechanical irritation of the inferior extremities of the divided nerves, similar effects to those produced by galvanism are occasioned, but in a weaker degree.

6to. The chief function of the pneumo-gastric nerves, considered in relation to digestion, is to preside over the movements of the stomach— movements which accelerate digestion, by facilitating the contact of the gastric juice with the different parts of the alimentary mass.

*Remarks.* These conclusions, after all, differ more in words than in things, from those to which Dr. Philip came. He concluded that galvanism supplied the place of the nervous influence, and thus excited the stomach to digestion—a process that was stopped, or nearly so, when the source of nervous influence was cut off by section of the eighth pair. We believe Dr. Philip never meant to insist on the *identity* of the nervous influence and galvanic fluid, from these experiments. In the present experiments the results are the same—the *modus operandi* or explanation of the phenomena differ. The Parisian experimenters look on the galvanic fluid as merely the most powerful excitant of the nerves, and through the nerves, of the muscular fibres of the stomach. This is probably the most correct view of the case, and is strongly countenanced by the experiments of producing a digestive process, however weak, by means of mechanical stimulus to the lower extremities of the divided nerves.

---

3. *Semi-decussation of the Optic Nerves.** Dr. Wollaston has been led to some anatomico-physiological speculations respecting the decussation of the optic nerves, from a few instances of disordered vision occurring in himself and others. He doubts the received opinion of the said decussation in man, and concludes that the distribution is otherwise.

"It is now more than twenty years since I was first affected with the peculiar state of vision to which I allude, in consequence of violent exercise I had taken for two or three hours before. I suddenly found that I could see but half the face of a man whom I met; and it was the same with respect to every object I looked at. In attempting to read the name *Johnson*, over a door, I saw only *son;* the commencement of the name being wholly obliterated to my view. In this instance the loss of sight was towards my left, and was the same whether I looked with the right eye or the left. This blindness was not so complete as to amount to absolute blackness, but was a shaded darkness without definite outline. The complaint was of short duration, and in about a quarter of an hour might be said to be wholly gone; having receded with a gradual motion from the centre of vision obliquely upwards toward the left.

"Since this defect arose from over-fatigue, a cause common to many other nervous affections, I saw no reason to apprehend any return of it, and it passed away without need of remedy, without any further explanation, and without my drawing any useful inference from it.

"It is now about fifteen months since a similar affection occurred again to myself, without my being able to assign any cause whatever, or to connect it with any previous or subsequent indisposition. The blindness was first observed, as before, in looking at the face of a person I met, whose left eye was to my sight obliterated. My blindness was in this instance the reverse of the former, being to my right (instead of the left) of the spot to which my eyes were directed; so that I have no reason to suppose it in any manner connected with the former affection.

---

* Dr. Hyde Wollaston, Philosoph. Trans 1824.

"The new punctum cæcum was situated alike in both eyes, and at an angle of about three degrees from the centre; for, when any object was viewed at the distance of about five yards, the point not seen was about ten inches distant from the point actually looked at.

"On this occasion the affection, after having lasted with little alteration for about twenty minutes, was removed suddenly and entirely, by the excitement of agreeable news respecting the safe arrival of a friend from a very hazardous enterprise."

Reflecting on this subject, Dr. Wollaston came to what he considers a probable interpretation of a set of facts which are not consistent with the generally received hypothesis of the decussation of the optic nerves. This interpretation we cannot abridge.

"Since the corresponding points of the two eyes sympathise in disease, their sympathy is evidently from structure, not from mere habit of feeling together, as might be inferred, if reference were had to the reception of ordinary impressions alone. Any two corresponding points must be supplied with a pair of filaments from the same nerve, and the seat of a disease in which similar parts of both eyes are affected must be considered as situated at a distance from the eyes, at some place in the course of the nerves where these filaments are still united, and probably in one or the other thalamus nervorum opticorum.

"It is plain that the cord, which comes finally to either eye, under the name of optic nerve, must be regarded as consisting of two portions,—one half from the right thalamus, and the other from the left thalamus nervorum opticorum.

"According to this supposition, decussation will take place only between the adjacent halves of the two nerves. That portion of nerve which proceeds from the right thalamus to the right side of the right eye, passes to its destination without interference; and, in a similar manner, the left thalamus will supply the left side of the left eye with one part of its fibres, while the remaining halves of both nerves, in passing over to the eyes of the opposite side, must intersect each other, either with or without intermixture of their fibres.

"Now, if we consider rightly the facts discovered by comparative anatomy in fishes, we shall find that the crossing of the entire nerves in them to the opposite eyes, is in perfect conformity to this view of the arrangement of the human optic nerves. The relative position of the eyes to each other in the sturgeon, is so exactly back to back, on opposite sides of the head, that they can hardly see the same object; they can have no points which generally receive the same impressions, as in us; there are no corresponding points of vision requiring to be supplied with fibres from the same nerve. The eye which sees to the left has its retina solely upon its right side, and this is supplied with an optic nerve arising wholly from the right thalamus; while the left thalamus sends its fibres entirely to the left side of the right eye, for the perception of objects situated on the right. In this animal, an injury to the left thalamus might be expected to occasion entire blindness of the right eye alone, and want of perception of objects placed on that side. In ourselves, a similar injury to the left thalamus would occasion blindness (as before) to all objects situated to our right, owing to insensibility of the left half of the retina of both eyes." 393.

Some other instances of half-blindness have fallen under the notice of Dr. Wollaston, and he suspects that the complaint is much more frequent than one would suppose from the absence of published documents.

P. S.—Dr. Wollaston appears to have been anticipated anatomically—or what is better, his physiological speculations appear to be confirmed by observations made by Vicq. D'Azyr, the Wenzels, and by M. Treviranus. We refer to the Journal Complémentaire of Oct. 1823, for particulars, but shall here give the following extract from a cotemporary Journal of that period.

"*Vicq D'Azyr* had found, on examining with a microscope, a horizontal section of the optic nerves of the human subject, after it had been hardened by immersion in alcohol; that the medullary fibres, occupying the exterior side of the optic nerve, proceed in a direct manner from the optic thalamus to the eye of the same side; and that the place of union presents an homogeneous tissue. The *Wenzels* came nearly to a similar conclusion from their observations, but remarked, in addition, that while the fibres of the exterior side of the nerve go immediately to the eye of the same side, those fibres, placed in its interior side, are directed obliquely towards the other nerve, without, however, any crossing of fibres being manifest at the point where the junction of both nerves takes place.

"*M. Treviranus* has, in a great measure, confirmed these observations, on the male *simia aygula*. The nerves and brain were left during some months in alcohol, and afterwards kept some time in caustic potash to soften them. Having thus prepared them, he submitted them to a careful dissection, when he made out, with the aid of a microscope, that the external fibres of the upper side of each were continued from their cerebral extremity to that in the eye, without uniting themselves to those of the other side; whilst, on the contrary, the internal and inferior fibres of one nerve went to the other side, and united with the fibres of the opposite nerve. It was difficult to determine whether any of the fibres actually passed from one side to the other. He thought, however, that some of the fibres did so. The internal fibres, thus interlacing together, were evidently more numerous than the external fibres which ran to the eye without uniting with those of the opposite nerve.*

4. *Partial Paralysis—Portio Dura.*† The observations of Mr. Bell on the nerves of the face are now confirmed by every day's experience.

* Vide London Med. Repos. for January, 1824.

† M. Billard, Archives Generales, November, 1824.

In the following case, while there is a confirmation, there is also a curious and puzzling anomaly, which we shall leave Mr. Bell to explain.

*Case.* A woman, 60 years of age, had a tumour in the region of the right parotid gland, which broke, and continued open for a long time, discharging peculiarly fetid pus. The patient also became affected with symptoms of pulmonary consumption. In the course of the disease, and in addition to the affection of the lungs, she shewed a peculiar change in the features which attracted particular attention. The right side of the face became paralyzed, but in a gradual manner, and with this exception that both the eyeball and upper eyelid of that side enjoyed their full power of motion, while the inferior eyelid was paralyzed and hung downwards, the lining membrane (conjunctiva) of which, was red and tumid. The eye itself was in a constant state of lachrymation. The nose was drawn to the opposite side. The right nostril was contracted—the left dilated. The mouth was remarkably distorted—the right side of the lips being pendulous and relaxed—the left drawn upwards and towards the ear of that side. The muscles of the tongue were unaffected. When the patient spoke or laughed, the face was hideously and ridiculously distorted. The right side of the face was without expression, and the features as it were dead, while the muscular action on the other side, being strongly developed, gave to this part of the face a most remarkable mobility and physiognomy. In sleep the upper eyelid covered its proper proportion of the eyeball, but the lower hung down. The sensibility of the paralyzed part of the face was as perfect as on the other side. At length the sore cicatrized, leaving a deep eschar, but the paralysis remained—she became hectic—and died phthisical. On dissection, scarcely any part of the parotid gland could be detected. The portio dura of the seventh pair was found to be destroyed, at its exit from the foramen stylo-mastoideum. "Je disséquai la portion dure de la septième paire à travers le rocher; elle se montra saine jusqu'à la sortie par le trou stylo-mastoidien; là commençait son interruption, de sorte qu'il manquait au nerf facial une portion de son trouc d'une longueur égale à la largeur de l'echancrure parotidienne."

"This case," says the author, "appears completely to confirm the observations and experiments of Mr. Charles Bell on the facial nerve." The anomaly in the motion of the upper eyelid remains to be explained. Dr. Bellard mentions several cases of partial paralysis of the face, in confirmation of Mr. Bell's doctrines, of which we shall only notice the following, related by M. Guessin, a distinguished physician of Angers. P. Chollet, aged 30 years, became affected in the month of December, 1821, with phlegmonous erysipelas of the face eight days after her confinement. Delirium supervened, but at length the erysipelas subsided, leaving violent pulsating pains in the mastoid region of the right side. There was some deafness, and a purulent discharge from the ear. It was at this time that the patient's mouth was found to be drawn to one side—the right eye remained open—the upper lip, and also the alæ nasi were drawn to the opposite side. There was no paralysis in any other part of the body. The sensibility was equally perfect in both sides of the face. The discharge from the ear ceased, but the paralysis and deformity remained, in spite of every remedy that could be employed. While tranquil, the face does not appear much distorted, but the moment that she attempts to speak or laugh, the deformity is great. The right eye is always in a state of lachrymation.

We sometime ago attended a medical gentleman who met with this accident. In coming up from Edinburgh, on his way to Italy, he caught cold in his right ear, and had some deep-seated pain there for some days. One morning, on getting out of bed, at his lodgings in St. Martin's-lane, he was alarmed at seeing his mouth drawn entirely to the left side. He sent for the Editor of this Journal, who easily recognized a compression of the portio dura, and assured Dr. M. that there was no affection of the brain. Mr. Shaw was invited to see the case, and immediately ascertained its nature. The sensibility was perfect in both sides of the face. Dr. M. went to the continent in this state, but ultimately recovered the power of the facial muscles, while in Italy. We may here remark that, in Dr. M.'s case, as in the first case related in the present paper, the motion of the upper eyelid was not lost—nor that of the lower. Dr. M. could just bring the eyelids together, but not closely. A slight line of the white of the eye could be seen when he was asleep. Some trifling degree of lachrymation was perceptible in that eye. In these affections then, of the portio dura, in man, there does not seem to be so much danger of the eye, as results when experiments are made on animals. The eye of the affected side remaining open in them, the conjunctiva inflames, and opacity of the cornea is the consequence. This does not appear likely to happen in the human species, independently of the power which man would have of closing the eye mechanically, and thus guarding it against the accident alluded to above.

